# The Central Role of the AMPK/SIRT1/PGC-1α Signaling Axis in Skeletal Muscle Physiology and Pathology and Its Targeted Therapeutic Strategies

**DOI:** 10.3390/ph19071056

**Published:** 2026-07-08

**Authors:** Jie Wang, Jiayi Gu, Xia Li, Hualin Sun, Xiaoming Yang

**Affiliations:** Jiangsu Key Laboratory of Tissue Engineering and Neuroregeneration, Key Laboratory of Neuroregeneration of Ministry of Education, Co-Innovation Center of Neuroregeneration, Medical School of Nantong University, Nantong University, Nantong 226001, China; 2022310019@stmail.ntu.edu.cn (J.W.); gujiayi0628@stmail.ntu.edu.cn (J.G.); 2324310018@stmail.ntu.edu.cn (X.L.); sunhl@ntu.edu.cn (H.S.)

**Keywords:** skeletal muscle, mitochondrial biogenesis, muscle atrophy, metabolic regulation, therapeutic strategies

## Abstract

Considered by some to be the largest metabolic organ of the body, the functional integrity of skeletal muscle is highly dependent on its exceptional plasticity, which is primarily governed by mitochondrial quality control. The signaling axis composed of AMP-activated protein kinase (AMPK), sirtuin 1 (SIRT1), and peroxisome proliferator-activated receptor gamma coactivator 1-alpha (PGC-1α) serves as a critical hub that senses cellular energy status, coordinates mitochondrial biogenesis, regulates muscle fiber type switching, and maintains protein homeostasis. This review systematically delineates the structural functions and synergistic regulatory network of the AMPK/SIRT1/PGC-1α signaling axis. It further elucidates the regulatory roles of this pathway under physiological conditions—such as exercise adaptation and muscle fiber-type transformation—and its dysregulated mechanisms in the pathogenesis of various skeletal muscle disorders, including sarcopenia, disuse atrophy, cachexia, neurogenic atrophy, muscular dystrophy, and type 2 diabetes mellitus-related myopathy. Building on this foundation, this review critically analyzes current multifaceted therapeutic strategies targeting this pathway, encompassing exercise and physical therapy, nutritional and natural products, and small molecule drugs, as well as gene and cell-based therapies. Finally, this review delves into the challenges facing clinical translation in this field, such as the complexity of the signaling network, individual variability, and bioavailability issues. It also proposes future research directions focused on developing precision intervention tools, establishing effective biomarker systems, and exploring combination intervention strategies. Collectively, the AMPK/SIRT1/PGC-1α signaling axis is central to maintaining skeletal muscle metabolic homeostasis, and targeting this pathway provides a robust theoretical foundation and broad application prospects for the prevention and treatment of skeletal muscle-related diseases.

## 1. Introduction

Skeletal muscle is not only a core component of the locomotor system but is also considered by some authors to be the body’s largest metabolic and endocrine organ, accounting for approximately 40–50% of the total protein reserve [1,2,3]. Through the efficient uptake and utilization of energy substrates such as glucose and fatty acids, skeletal muscle plays a decisive role in maintaining whole-body energy homeostasis [4]. Its functional integrity is highly dependent on its remarkable plasticity—the ability to dynamically adjust its metabolic characteristics, contractile performance, and myofiber type composition in response to internal and external environmental changes, including nutritional status, exercise load, and disease stress [5]. This intricate plasticity serves as the fundamental cornerstone for preserving muscle health, ensuring physical performance, and maintaining quality of life. Therefore, an in-depth understanding of the regulatory mechanisms governing skeletal muscle plasticity is essential for elucidating its physiological functions.

Mitochondria, functioning as the cell’s “energy factories,” are particularly abundant in skeletal muscle, and their functional state directly determines muscle metabolic capacity and endurance. The mitochondrial quality control system, comprising mitochondrial biogenesis, dynamics (fusion/fission), and mitophagy, constitutes the core mechanism by which skeletal muscle maintains its metabolic homeostasis [4]. However, under pathological conditions such as aging, chronic diseases, malnutrition, or disuse, mitochondrial dysfunction occurs, leading to disrupted energy metabolism, excessive reactive oxygen species production, and exacerbated inflammatory responses, ultimately triggering muscle atrophy and functional impairment [4,6]. Consequently, a thorough understanding of the regulatory mechanisms of mitochondrial quality control is critical for the prevention and treatment of skeletal muscle-related disorders. In light of this, targeting mitochondrial function has emerged as a key therapeutic strategy for intervening in muscle atrophy.

Among the numerous signaling pathways that regulate mitochondrial function and muscle metabolism, the AMPK/SIRT1/PGC-1α axis plays a central hub role. AMP-activated protein kinase (AMPK) serves as the most sensitive energy sensor within the cell, becoming activated upon a decline in ATP levels to restore energy balance [7,8]. Concurrently, sirtuin 1 (SIRT1), a NAD^+^-dependent histone deacetylase, acts as a sensitive sensor of the cellular energy and redox state [9]. These two factors work synergistically to co-activate peroxisome proliferator-activated receptor gamma coactivator 1-alpha (PGC-1α). PGC-1α is a master regulator of mitochondrial biogenesis and oxidative metabolism; by co-activating a cascade of downstream transcription factors (such as NRF1, NRF2, ERRα, and PPARs), it promotes mitochondrial DNA replication, the expression of electron transport chain complexes, fatty acid oxidation, and the production of antioxidant enzymes, thereby shaping a muscle phenotype characterized by high oxidative capacity and high energy efficiency [10,11]. Notably, the activity of this pathway is finely regulated by diverse physiological and pathological signals, and its dysregulation is closely associated with the onset and progression of various skeletal muscle diseases [12]. Thus, elucidating the regulatory networks governing this pathway under both physiological and pathological conditions holds significant research value.

In recent years, a substantial body of research, spanning from foundational exercise science to advanced molecular pharmacology, has confirmed the critical role of the AMPK/SIRT1/PGC-1α signaling pathway in multiple skeletal muscle disorders and has highlighted its immense potential as a therapeutic target. For instance, in age-related sarcopenia, decreased activity of this pathway contributes to mitochondrial dysfunction and loss of muscle mass [13]; whereas in type 2 diabetes-associated myopathy, impairment of this pathway exacerbates insulin resistance and metabolic dysregulation [14]. These findings not only deepen our understanding of disease mechanisms but also provide a theoretical foundation for developing targeted intervention strategies.

This review will systematically delineate the central regulatory role of the AMPK/SIRT1/PGC-1α signaling axis in skeletal muscle physiology (e.g., exercise adaptation, myofiber type transformation) and pathology (e.g., sarcopenia, cachexia, diabetic myopathy). Building upon this, this article will thoroughly analyze the current challenges associated with targeting this pathway for therapeutic intervention, with a focus on exploring diverse treatment strategies, including exercise, natural products, small-molecule compounds, and gene therapy. This analysis aims to provide novel perspectives and a theoretical basis for the prevention and treatment of skeletal muscle diseases. Through an integrated analysis of existing research, this review aspires to offer directional guidance for the future development of safer and more effective therapeutic approaches for muscle disorders.

## 2. Literature Search Strategy

To ensure transparency and reproducibility in this narrative review, we explicitly detail the literature search methodology here. A structured search was conducted in PubMed and Web of Science for articles published up to June 2026. The Boolean search strategy used was: (“AMPK” OR “AMP-activated protein kinase”) AND (“SIRT1” OR “Sirtuin 1”) AND (“PGC-1α” OR “PGC1A”) AND (“skeletal muscle” OR “myopathy” OR “sarcopenia” OR “muscle atrophy” OR “cachexia” OR “muscular dystrophy” OR “insulin resistance”). We included only peer-reviewed original research articles and reviews published in English, while excluding conference abstracts, preprints, and off-topic studies. Reference lists of key reviews were additionally hand-searched. Article selection was guided by relevance and significance, not by a formal quantitative scoring system.

## 3. AMPK/SIRT1/PGC-1α Signaling Axis: Structural and Functional Insights

**Figure 1:**
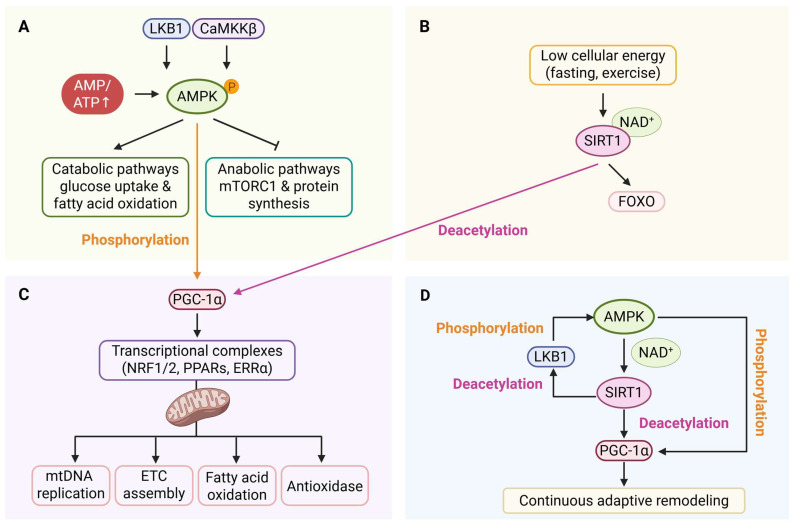
Structural and functional overview of the AMPK/SIRT1/PGC-1α signaling axis. Panel (**A**): AMPK heterotrimeric complex (α, β, γ subunits) with upstream kinases LKB1 and CaMKKβ. Panel (**B**): SIRT1 with NAD^+^ and its substrates PGC-1α and FOXO. Panel (**C**): PGC-1α coactivating downstream transcription factors (NRF1/2, PPARs, ERRα) to regulate mitochondrial biogenesis, fatty acid oxidation, and antioxidant defense. Panel (**D**): Positive feedback loop among AMPK, SIRT1, and PGC-1α. Arrows indicate direct/indirect regulation; T-bars inhibit; colors distinguish pathways. Abbreviations: AMPK, AMP-activated protein kinase; SIRT1, sirtuin 1; PGC-1α, peroxisome proliferator-activated receptor gamma coactivator 1-alpha; LKB1, liver kinase B1; CaMKKβ, calcium/calmodulin-dependent protein kinase kinase β; ATP, adenosine triphosphate; AMP, adenosine monophosphate; mTORC1, mechanistic target of rapamycin complex 1; NAD^+^, nicotinamide adenine dinucleotide; FOXO, forkhead box O; NRF1/2, nuclear respiratory factor 1/2; PPARs, peroxisome proliferator-activated receptors; ERRα, estrogen-related receptor alpha; mtDNA, mitochondrial DNA; ETC, electron transport chain. Created in BioRender. Jie, W. (2026) https://BioRender.com/5wl17h0.

Skeletal muscle, as a highly plastic tissue, relies on a sophisticated signaling network to maintain metabolic homeostasis. Among these, the signaling axis composed of AMP-activated protein kinase (AMPK), sirtuin 1 (SIRT1), and peroxisome proliferator-activated receptor gamma coactivator 1-alpha (PGC-1α) serves as a central regulatory hub for cellular energy metabolism (Figure 1), playing an irreplaceable role in sensing energy fluctuations, coordinating catabolic and anabolic processes, and driving mitochondrial biogenesis [15,16]. A comprehensive understanding of the structure, function, and intricate crosstalk within this axis is critical for elucidating the molecular basis of skeletal muscle physiological adaptation and pathological alterations, thereby establishing it as a key therapeutic target for metabolic diseases such as muscle atrophy.

### 3.1. AMPK: The Master Energy Sensor

AMPK is a heterotrimeric protein complex, comprising a catalytic α subunit and regulatory β and γ subunits [12]. Ubiquitously expressed in eukaryotic cells, it is widely recognized as the “master switch” of cellular energy metabolism. Its activation mechanism is highly conserved: a decline in cellular energy status, reflected by decreased ATP/AMP and ATP/ADP ratios, leads to competitive binding of AMP and ADP to the CBS (cystathionine beta-synthase) domains on the γ subunit. This induces a conformational change that facilitates phosphorylation of the α subunit at Thr172 by upstream kinases, primarily liver kinase B1 (LKB1) and calcium/calmodulin-dependent protein kinase kinase β (CaMKKβ), resulting in robust AMPK activation [4,11,17]. Beyond energy stress, a diverse array of physiological stimuli (such as exercise and fasting), pathological conditions (including oxidative stress and ischemia/hypoxia), and various pharmacological agents (e.g., metformin) can also activate AMPK, underscoring its central role in integrating diverse intra- and extracellular signals [18,19]. Therefore, AMPK serves as a critical hub linking cellular energy status to broad metabolic and stress-responsive pathways, ultimately maintaining cellular homeostasis.

In skeletal muscle, AMPK activation serves as a critical switch that initiates adaptive metabolic responses. Upon activation, AMPK rapidly shuts down energy-consuming anabolic pathways by phosphorylating downstream effectors—for instance, inhibiting acetyl-CoA carboxylase (ACC) to reduce fatty acid synthesis and suppressing mTORC1 to decrease protein synthesis. Concurrently, it promotes energy-producing catabolic pathways, facilitating glucose uptake (via GLUT4 translocation) and fatty acid oxidation to meet immediate energy demands [11,15]. Notably, AMPK activation constitutes one of the initiating steps in the mitochondrial biogenesis program. It drives adaptive mitochondrial remodeling by directly phosphorylating or indirectly modulating PGC-1α, thereby enhancing the oxidative capacity and fatigue resistance of skeletal muscle [17,18]. Consequently, AMPK functions not only as an instantaneous sensor of energy status but also as a crucial bridge connecting energy metabolism to long-term adaptive remodeling, with its functional integrity being essential for maintaining skeletal muscle health.

### 3.2. SIRT1: An NAD^+^-Dependent Deacetylase

SIRT1 is the most extensively characterized member of the sirtuin family, and its enzymatic activity is strictly dependent on the coenzyme nicotinamide adenine dinucleotide (NAD^+^). Consequently, SIRT1 functions not only as a sensor of cellular energy status but also as an indicator of the cellular redox state. Under conditions of low cellular energy, such as fasting or exercise, NAD^+^ levels rise, leading to enhanced SIRT1 activity. This mechanism tightly couples the cellular energy and redox status with transcriptional regulatory programs [9,11]. Through the deacetylation of a diverse array of substrates, including histones, transcription factors, and coactivators, SIRT1 is intricately involved in the fine-tuning of various processes, such as cellular metabolism, apoptosis, senescence, and stress responses. Thus, SIRT1 plays a pivotal role in determining cell fate.

In skeletal muscle, the activation of SIRT1 is closely associated with the pleiotropic benefits conferred by exercise training. A key underlying mechanism is the direct interaction of SIRT1 with PGC-1α, leading to its deacetylation—a critical rate-limiting step for activating PGC-1α transcriptional activity [9]. Furthermore, SIRT1 can indirectly promote AMPK phosphorylation by deacetylating and activating LKB1, thereby establishing a positive feedback loop that amplifies and sustains energy stress signals [15,20]. Beyond its role in regulating energy metabolism, SIRT1 also deacetylates forkhead box O (FOXO) family transcription factors, which upregulates the expression of antioxidant enzymes (e.g., MnSOD, Catalase). This enhances the cellular antioxidant defense capacity, protecting skeletal muscle from oxidative stress-induced damage [19,21]. Therefore, SIRT1 not only orchestrates energy metabolism and mitochondrial function but also serves as a critical node linking metabolic status with cell survival signaling.

### 3.3. PGC-1α: The Master Regulator of Mitochondrial Biogenesis

PGC-1α is a transcriptional coactivator that lacks intrinsic DNA-binding capability. Instead, it forms transcriptional complexes by interacting with various nuclear receptors and transcription factors, such as PPARs, ERRα, and NRF1/2, thereby potently driving the expression of target genes. These target genes encompass virtually all aspects of mitochondrial biogenesis and function, including mitochondrial DNA (mtDNA) replication and transcription (via activation of NRF1/2 and TFAM), assembly of electron transport chain complexes, fatty acid oxidation (via activation of PPARα and ERRα), and the establishment of antioxidant defense systems (e.g., SOD2, GPx1) [10,18]. Owing to its integrative control over these core processes, PGC-1α is recognized as the “master regulator” of mitochondrial biogenesis, positioning it as a critical molecular hub for maintaining energy homeostasis in skeletal muscle.

The activity of PGC-1α is subject to precise, multi-layered regulation, with post-translational modifications mediated by AMPK and SIRT1 representing the primary mechanisms of its activation. Specifically, AMPK can directly phosphorylate PGC-1α, thereby enhancing its stability and transcriptional activity. Concurrently, SIRT1 deacetylates PGC-1α, relieving inhibitory acetylation modifications to fully unleash its coactivator potential [9,10,12]. Notably, stimuli such as exercise, cold exposure, and certain natural products (e.g., resveratrol) effectively upregulate the expression and activity of PGC-1α. This drives a phenotypic shift in skeletal muscle towards a more oxidative, fatigue-resistant profile, representing the core molecular basis for the beneficial effects of exercise on muscle function [19,22]. Consequently, a detailed understanding of the regulatory network governing PGC-1α is of great significance for comprehending and therapeutically intervening in muscle functional decline.

### 3.4. Tripartite Crosstalk: Synergy and Feedback

AMPK, SIRT1 and PGC-1α do not operate in a simple linear pathway; instead, they form a highly integrated, synergistic regulatory network that includes a positive feedback loop [11,15,18]. Firstly, AMPK and SIRT1 act in concert to co-activate PGC-1α, thereby initiating downstream mitochondrial biogenesis. Specifically, AMPK primes PGC-1α through phosphorylation, whilst simultaneously enhancing SIRT1 activity by increasing intracellular NAD^+^ levels—for instance, via activation of nicotinamide phosphoribosyltransferase (NAMPT). Subsequently, SIRT1 reinforces PGC-1α activity through deacetylation; this dual regulatory mechanism ensures maximal PGC-1α activation [15,18]. Secondly, a self-amplifying positive feedback loop exists within this signaling axis: SIRT1 activates LKB1 via deacetylation, which in turn promotes AMPK phosphorylation. Concurrently, activated AMPK feeds back to enhance SIRT1 activity by elevating NAD^+^ levels, thus forming a self-reinforcing positive feedback cycle that ensures a rapid and sustained adaptive metabolic remodeling in response to energy stress [19,20]. Collectively, the intricate coordination among these three molecules constitutes a central hub in the regulation of skeletal muscle energy metabolism.

Furthermore, this signaling pathway closely interacts with other key signaling cascades—such as the insulin/PI3K/Akt, FOXO, and NF-κB pathways—to collectively determine the fate of skeletal muscle under conditions of nutritional variation, energy stress, and other physiological challenges. For example, under nutrient-rich conditions, the Akt/mTOR pathway predominates to promote protein synthesis and muscle growth. Conversely, under energy stress or in disease states, the AMPK/SIRT1/PGC-1α axis takes precedence, favoring catabolic processes to generate energy and initiating mitochondrial turnover to preserve cellular homeostasis [18,19]. In parallel, activation of this axis exerts cytoprotective effects against various pathological insults by enhancing antioxidant capacity and suppressing NF-κB-mediated inflammatory responses [18,21]. Thus, the dynamic balance and reciprocal regulation among these pathways collectively dictate the adaptive direction of skeletal muscle across different physiological and pathological contexts. Elucidating this central signaling axis and its crosstalk with other pathways provides a critical theoretical foundation for understanding skeletal muscle regulation at the molecular level, as well as for developing targeted intervention strategies. Future research focused on dissecting these complex interaction networks holds promise for identifying novel therapeutic avenues for muscle-wasting disorders.

Beyond the positive feedback loop described above, the AMPK/SIRT1/PGC-1α axis also engages in extensive inhibitory crosstalk with other signaling cascades. For instance, under nutrient-rich conditions, the Akt/mTOR pathway predominates to promote protein synthesis and muscle growth, whereas AMPK activation suppresses mTORC1 activity to conserve energy. Similarly, activation of AMPK/SIRT1/PGC-1α can antagonize NF-κB-mediated inflammatory responses, thereby protecting skeletal muscle from chronic inflammation-induced catabolism. These context-dependent interactions are further elaborated in Section 5.1. Understanding this dynamic balance is essential for designing interventions that selectively activate the desired effects without disrupting overall cellular homeostasis.

## 4. The Role of the AMPK/SIRT1/PGC-1α Pathway in Skeletal Muscle Physiology and Pathology

The AMPK/SIRT1/PGC-1α signaling axis serves as a central regulatory network governing metabolic homeostasis in skeletal muscle. Functioning as a sophisticated signaling hub, this pathway integrates diverse upstream signals—including cellular energy status (indicated by the AMP/ATP ratio), NAD^+^ levels (reflecting the cellular redox state), and calcium signaling—to precisely orchestrate skeletal muscle energy metabolism, mitochondrial biogenesis, muscle fiber type switching, and protein homeostasis. It achieves this by coordinating dynamic changes in gene expression and protein function, thereby enabling precise adaptation to various physiological demands (such as exercise and fasting) and mounting effective responses to pathological stimuli. Consequently, an in-depth understanding of the regulatory mechanisms governing this pathway is of great significance for elucidating skeletal muscle physiology and developing interventions for related diseases.

### 4.1. Regulatory Roles Under Physiological Conditions

#### 4.1.1. Exercise Adaptation and Energy Metabolism

Exercise, particularly endurance exercise, represents the most potent and extensively studied physiological stimulus for activating the AMPK/SIRT1/PGC-1α pathway [8,17]. Specifically, the depletion of ATP during exercise elevates the AMP/ATP ratio, while concurrent Ca^2+^ release from the sarcoplasmic reticulum rapidly activates AMPK and CaMKKβ, respectively. These events subsequently lead to the phosphorylation and activation of PGC-1α, initiating downstream transcriptional programs [17]. Furthermore, long-term exercise training, through repeated activation of this pathway, not only significantly promotes skeletal muscle mitochondrial biogenesis and increases capillary density but also markedly enhances the capacity of skeletal muscle to utilize fatty acids for energy by upregulating the expression of genes involved in fatty acid oxidation [23]. Notably, exercise-induced adaptive changes are not confined to intracellular events but also involve autocrine and paracrine regulation by myokines. For instance, exercise upregulates the expression of FNDC5 in skeletal muscle; its cleaved product, irisin, is secreted into the circulation. Irisin can not only feedback-regulate muscle metabolism, regeneration, and mitochondrial function in an autocrine/paracrine manner but also activate the AMPK/SIRT1/PGC-1α pathway itself, thereby establishing a positive regulatory loop that amplifies mitochondrial biogenesis [24]. Moreover, these exercise-induced adaptive changes are further amplified by a network of myokines, which function as pleiotropic signaling modulators orchestrating inter-organ crosstalk and systemic metabolic benefits [5]. Concurrently, mild heat stress generated during exercise has been demonstrated to be sufficient to activate this pathway and promote mitochondrial biogenesis, representing another important mechanism through which exercise improves muscle health [25]. Collectively, these findings reveal a complex network through which exercise achieves multi-level, multi-target regulation of muscle metabolic adaptation via the AMPK/SIRT1/PGC-1α axis.

#### 4.1.2. Muscle Fiber Type Switching

The remarkable plasticity of skeletal muscle fibers underpins their adaptation to diverse functional demands, with PGC-1α being recognized as a key determinant of muscle fiber type. Experimental evidence demonstrates that overexpression of PGC-1α is sufficient to convert fast-twitch glycolytic (type IIb/x) muscle fibers into more oxidative, slow-twitch (type I) and fast-twitch oxidative-glycolytic (type IIa) fibers [23]. In this process, the AMPK/SIRT1 pathway plays a central role in regulating fiber type switching by modulating both the transcriptional activity and protein stability of PGC-1α [22]. For example, resveratrol, a natural SIRT1 activator, effectively promotes the transition from fast-to-slow-twitch fibers in C2C12 myotubes by suppressing the expression of miR-22-3p, thereby relieving its inhibitory effect on the AMPK/SIRT1/PGC-1α pathway [22]. Conversely, overexpression of miR-22-3p inhibits this pathway and promotes a fast-twitch fiber phenotype [23]. Beyond pharmacological interventions, nutritional factors also profoundly participate in this regulatory process. Research indicates that dietary leucine and fish oil can synergistically activate the CaMKII signaling pathway, subsequently upregulating the expression of myocyte enhancer factor 2C (MEF2C) and key components of this pathway (AMPK, SIRT1, PGC-1α), collectively promoting the conversion towards oxidative muscle fibers [26]. These findings highlight the substantial potential of nutrient combinations to remodel muscle fiber type by modulating this signaling axis.

#### 4.1.3. Balance Between Protein Synthesis and Degradation

**Figure 2:**
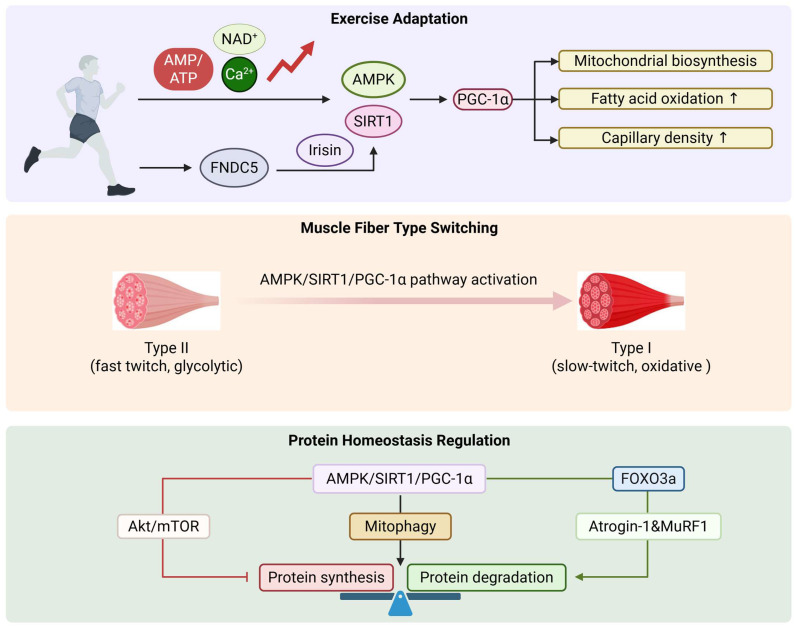
Physiological roles of the AMPK/SIRT1/PGC-1α axis in skeletal muscle. Left panel: Endurance exercise increases NAD^+^, AMP/ATP ratio, and Ca^2+^, activating AMPK and SIRT1 to promote mitochondrial biogenesis, fatty acid oxidation, and capillary density; irisin (cleaved from FNDC5) forms a positive feedback loop. Middle panel: Pathway activation drives conversion from fast-twitch glycolytic (Type II) to slow-twitch oxidative (Type I) fibers. Right panel: Pathway balances protein synthesis (via Akt/mTOR inhibition) and degradation (via FOXO3a upregulation of Atrogin-1 and MuRF1), while promoting mitophagy. Arrows indicate direct/indirect regulation; T-bars inhibit; colors distinguish pathways. Abbreviations: AMPK, AMP-activated protein kinase; SIRT1, sirtuin 1; PGC-1α, peroxisome proliferator-activated receptor gamma coactivator 1-alpha; NAD^+^, nicotinamide adenine dinucleotide; ATP, adenosine triphosphate; AMP, adenosine monophosphate; Ca^2+^, calcium ion; FNDC5, fibronectin type III domain-containing protein 5; Akt, protein kinase B; mTOR, mechanistic target of rapamycin; FOXO3a, forkhead box O3a; MuRF1, muscle RING finger 1. Created in BioRender. Jie, W. (2026) https://BioRender.com/lcj1cvr.

The AMPK/SIRT1/PGC-1α pathway is not only central to metabolic regulation but also deeply involved in maintaining protein homeostasis in skeletal muscle, acting as both an “energy sensor” and a “homeostasis regulator.” Under conditions of energy deficiency or catabolic stress, activation of this pathway reduces protein synthesis, primarily by inhibiting the Akt/mTOR pathway. Concurrently, it promotes protein degradation by activating transcription factors such as FOXO3a, leading to the upregulation of the muscle-specific E3 ubiquitin ligases Atrogin-1 and MuRF1, thereby mobilizing amino acids for energy production [15,18]. However, this enhanced catabolic state can be pathologically amplified under conditions such as aging, cachexia, or long-term glucocorticoid therapy, resulting in muscle atrophy [27]. Importantly, moderate levels of autophagy and mitophagy are crucial for clearing damaged proteins and organelles to maintain muscle homeostasis, a process also finely regulated by the AMPK/SIRT1/PGC-1α pathway [28,29]. Studies have shown that this pathway plays a critical role in maintaining muscle mass and function by regulating mitophagy [29] (Figure 2). Therefore, by integrating energy metabolism, protein synthesis/degradation, and organelle quality control, the AMPK/SIRT1/PGC-1α pathway plays an irreplaceable and central role in maintaining skeletal muscle homeostasis, serving as a critical hub for preserving muscle health.

### 4.2. Dysregulation and Pathological Roles

#### 4.2.1. Muscle Atrophy Disorders

**Figure 3:**
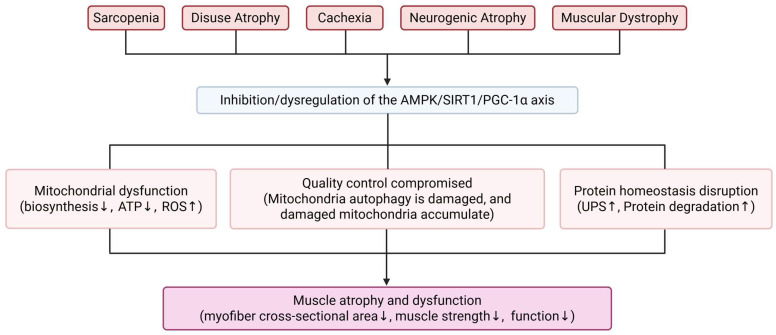
Dysregulation of the AMPK/SIRT1/PGC-1α axis in skeletal muscle pathologies. Various pathological conditions (sarcopenia, disuse atrophy, cachexia, neurogenic atrophy, muscular dystrophy) converge to inhibit/dysregulate the axis, leading to three core consequences: (1) mitochondrial dysfunction (decreased biogenesis, reduced ATP, increased ROS); (2) compromised quality control (impaired mitophagy, accumulation of damaged mitochondria); (3) disrupted protein homeostasis (UPS activation, increased protein degradation). These cellular defects ultimately result in muscle atrophy and functional decline (reduced fiber cross-sectional area, loss of strength). Abbreviations: AMPK, AMP-activated protein kinase; SIRT1, sirtuin 1; PGC-1α, peroxisome proliferator-activated receptor gamma coactivator 1-alpha; ATP, adenosine triphosphate; ROS, reactive oxygen species; UPS, ubiquitin-proteasome system. Created in BioRender. Jie, W. (2026) https://BioRender.com/l7vxl18.

Muscle atrophy represents a common endpoint of various diseases, aging, and disuse states, with core pathological features characterized by a reduction in myofiber cross-sectional area, increased protein degradation, decreased muscle strength, and loss of function [1,30]. A substantial body of research has established that the inhibition or functional dysregulation of the AMPK/SIRT1/PGC-1α signaling axis constitutes a critical molecular event driving the initiation and progression of various muscle atrophy disorders. Inactivation of this pathway not only directly impairs mitochondrial biogenesis and function but also disrupts the homeostatic balance between intracellular energy metabolism and protein degradation, thereby exacerbating muscle degeneration [4] (Figure 3). Consequently, targeted activation of this signaling axis has emerged as a crucial strategy for intervening in muscle atrophy.

##### Sarcopenia

As a prototypical aging-related degenerative disease, the core pathological mechanism of sarcopenia is widely attributed to mitochondrial dysfunction [4]. Numerous studies have demonstrated that the expression levels and activities of AMPK, SIRT1, and PGC-1α are significantly reduced in the skeletal muscle of aged individuals [7,28]. This suppression of the signaling axis directly leads to decreased mitochondrial biogenesis, insufficient cellular energy supply (ATP), elevated oxidative stress, and impaired autophagic flux, which collectively culminate in exacerbated protein degradation, muscle atrophy, and functional decline [4]. Specifically, during aging, decreased NAD^+^ levels in skeletal muscle, reduced AMPK and SIRT1 activities, and decreased PGC-1α expression alongside increased acetylation collectively impair mitochondrial biogenesis and function [31]. Subsequent mitochondrial dysfunction further promotes the production of reactive oxygen species (ROS), activates the FOXO pathway, upregulates Atrogin-1 and MuRF1, and intensifies protein degradation, creating a vicious cycle [1]. Additionally, chronic low-grade inflammation (inflammaging) during aging suppresses AMPK/SIRT1/PGC-1α activity, including via the NF-κB pathway [4]. Therefore, inactivation of the AMPK/SIRT1/PGC-1α signaling axis is considered a key molecular event driving age-related muscle degeneration, and restoring the activity of this pathway has become a core strategy for combating sarcopenia.

Notably, activating this signaling pathway through exercise or various pharmacological interventions has been shown to effectively improve muscle mass and function in aged animals, thereby delaying the progression of sarcopenia. For instance, exercise interventions can restore mitochondrial homeostasis by activating the AMPK/SIRT1/PGC-1α pathway, thereby ameliorating muscle atrophy in aged zebrafish and rodents [32,33]. In terms of pharmacological interventions, the natural flavonoid Ampelopsin has been shown to alleviate D-galactose-induced muscle atrophy in aged rats by modulating the AMPK signaling pathway [28]. Similarly, the traditional Chinese medicine component berberine effectively ameliorates muscle dysfunction and cognitive deficits in naturally aged rats by activating the AMPK/SIRT1/PGC-1α pathway [31]. Resveratrol has also been shown to exert muscle-protective effects in aging models [7,34] (detailed mechanistic discussion in Section 5.2). Collectively, these findings indicate that targeted activation of the AMPK/SIRT1/PGC-1α signaling axis, whether through physiological stimulation or pharmacological intervention, is an effective strategy for ameliorating sarcopenia.

In recent years, a range of active ingredients derived from natural products or functional foods has shown potential for combating sarcopenia by modulating the AMPK/SIRT1/PGC-1α pathway. For example, nano-sized vesicles derived from goji berries (*Lycium barbarum* L.) (GqDNVs) significantly improve skeletal muscle mass and function by activating this signaling pathway [35]. Extracellular vesicles from Chinese chives (*Allium tuberosum*) (CL-EVs) improve sarcopenia by activating the AMPK/SIRT1/PGC-1α axis while simultaneously inhibiting the Akt/FoxO3a/Atrogin-1/MuRF1 protein degradation pathway, thus dually regulating muscle homeostasis [36]. Additionally, the natural soy-derived triterpenoid Soyasapogenol B can directly bind to and activate SIRT1, thereby enhancing PGC-1α-mediated mitochondrial biogenesis and effectively improving muscle mass and endurance [19]. Moreover, active molecules such as paeoniflorin, asiatic acid, and the andrographolide derivative A-1 have also been reported to successfully ameliorate skeletal muscle atrophy induced by chronic kidney disease, dexamethasone, or neurodegenerative diseases by activating the AMPK/SIRT1/PGC-1α axis or its downstream branches [18,27,37]. These discoveries not only confirm the substantial potential of natural products in regulating muscle homeostasis but also provide a rich source of candidate molecules for developing anti-sarcopenia functional foods or drugs targeting this pathway.

It is particularly noteworthy that the accumulation of mitochondrial DNA (mtDNA) mutations also plays a significant role in age-related sarcopenia. If damaged mitochondria are not efficiently cleared (mitophagy), they release more ROS, further damaging mtDNA and the AMPK/SIRT1 pathway, thereby creating a vicious ‘mitochondria-energy sensor’ cycle [38]. Therefore, solely activating PGC-1α to promote mitochondrial biogenesis without concurrently enhancing mitophagy could increase the accumulation of dysfunctional mitochondria, potentially proving counterproductive. Future therapeutic strategies may necessitate simultaneously targeting both ‘biogenesis’ and ‘quality control’ processes. Beyond this vicious cycle, several unresolved questions remain regarding the therapeutic targeting of this pathway. First, it is unclear whether chronic AMPK activation, while beneficial for mitochondrial biogenesis, may inadvertently promote excessive catabolism and protein degradation via prolonged FOXO activation, potentially worsening muscle mass over the long term. Second, the optimal “therapeutic window” for pathway activation likely varies depending on disease stage, age, and metabolic status, what is beneficial in early sarcopenia might be ineffective or even harmful in advanced disease. Third, a fundamental but often overlooked issue is the need to balance mitochondrial biogenesis with quality control mechanisms. Simply driving PGC-1α-mediated biogenesis without simultaneously enhancing the clearance of damaged organelles could lead to the accumulation of dysfunctional mitochondria, exacerbating oxidative stress and energy deficits. Therefore, future interventions should aim to restore both the quantity and the quality of the mitochondrial pool, rather than focusing exclusively on biogenesis.

These findings not only provide promising intervention strategies for the prevention and treatment of sarcopenia but also robustly corroborate the central role of the AMPK/SIRT1/PGC-1α signaling axis in maintaining skeletal muscle metabolic homeostasis, underscoring its core value as a key therapeutic target against sarcopenia. Future precise interventions targeting this pathway, including the development of novel activators and exploration of synergistic effects between different interventions (such as combining exercise with nutritional supplements), represent important research directions in this field.

##### Disuse Atrophy

Disuse atrophy refers to the process of skeletal muscle wasting resulting from reduced physical activity due to limb immobilization, prolonged bed rest, or microgravity conditions. The underlying pathophysiology is closely associated with marked suppression of the AMPK/SIRT1/PGC-1α signaling axis. Specifically, prolonged inactivity leads to a sharp decline in the energy demand of skeletal muscle, resulting in reduced AMPK activity and subsequent downregulation of PGC-1α expression. This directly impairs mitochondrial oxidative capacity and biogenesis, manifesting as mitochondrial dysfunction and disrupted energy metabolism [4]. Concurrently, this process is accompanied by excessive activation of protein degradation pathways, particularly the ubiquitin-proteasome system (UPS), which accelerates the breakdown of myofibrillar proteins through upregulation of muscle atrophy-related genes such as MuRF1 and Atrogin-1 [39,40]. Collectively, the functional decline of the AMPK/SIRT1/PGC-1α signaling axis represents a central mechanism in the development and progression of disuse atrophy [12].

Therefore, restoring the activity of the AMPK/SIRT1/PGC-1α pathway through nutritional supplementation or pharmacological intervention is considered an effective strategy to counteract disuse atrophy. To this end, extensive research has focused on identifying natural products or drugs capable of activating this signaling axis to alleviate muscle wasting. For instance, studies have shown that Tangshenoside I, an active component derived from *Codonopsis lanceolata*, effectively counteracts immobilization-induced muscle atrophy by activating the SIRT1/PGC-1α pathway [41]. Similarly, GqDNVs have been shown to improve skeletal muscle mass and function via this pathway (discussed in detail in Section 5.2) [35]. Moreover, paeoniflorin has been shown to ameliorate skeletal muscle atrophy in a chronic kidney disease model through pathway-mediated antioxidant effects and protection of mitochondrial function [18]. In addition, exercise—recognized as one of the most effective means to activate this pathway—and its mimetics, such as resveratrol combined with metformin, have been found to promote myogenesis via the same axis [24]. Collectively, these studies not only provide robust experimental evidence for developing interventions against disuse atrophy but also collectively highlight the broad therapeutic potential of targeting the AMPK/SIRT1/PGC-1α axis.

##### Cachexia

Cachexia, occurring in the context of chronic diseases such as cancer, heart failure, and chronic kidney disease (CKD), involves a process of muscle wasting that is often accompanied by severe systemic inflammation and closely associated with mitochondrial dysfunction. These factors mutually reinforce each other, creating a vicious cycle [4,42]. In parallel, in chronic disease states such as cancer cachexia, persistently elevated IL-6 acts as a key mediator of muscle atrophy via the downstream JAK/STAT pathway, representing another major therapeutic target that operates alongside the AMPK/SIRT1/PGC-1α axis [43]. In CKD models, muscle wasting is strongly linked to increased oxidative stress and impaired mitochondrial function [44]. Studies have found that paeoniflorin, a component derived from traditional Chinese medicine, can significantly alleviate muscle wasting in CKD model rats, improve mitochondrial function, and reduce oxidative stress levels by activating the AMPK/SIRT1/PGC-1α pathway [18]. Similarly, the activity of this pathway is suppressed in diabetes-induced muscle atrophy [45]. Notably, mitochondrial dysfunction represents a core element in the pathogenesis of muscle atrophy induced by these metabolic diseases, and targeting the AMPK/SIRT1/PGC-1α signaling axis to restore mitochondrial biogenesis and function has emerged as a highly promising interventional strategy [4,19]. Therefore, a thorough understanding of the regulatory mechanisms governing this signaling axis is of great significance for developing effective interventions against chronic disease-related muscle atrophy.

Of note, various natural products and their derivatives have demonstrated therapeutic potential in models of diabetic muscle atrophy. For instance, *Ganoderma lucidum* proteoglycan has been shown to ameliorate muscle atrophy in diabetic rats by activating the AMPK/SIRT1 pathway [45]. Furthermore, GqDNVs have been confirmed to effectively inhibit dexamethasone-induced myotube atrophy, with their mechanism also relying on the activation of the AMPK/SIRT1/PGC-1α pathway (see Section 5.2 for a detailed discussion) [35]. Additionally, luteolin has been shown in obesity and diabetes models to ameliorate mitochondrial dysfunction and inhibit protein degradation through the activation of this pathway, effectively improving muscle mass [46]. Collectively, these studies underscore the central regulatory role of the AMPK/SIRT1/PGC-1α pathway in metabolic muscle atrophy and suggest that natural products represent potentially effective activators of this pathway.

Beyond the aforementioned natural products, other naturally occurring compounds and their derivatives have also demonstrated potential for ameliorating metabolic muscle atrophy via this pathway. For example, cordycepin exerts anti-fatigue effects by activating the TIGAR/SIRT1/PGC-1α signaling pathway, indicating its potential value in improving muscle function [47]. Moreover, an aqueous extract from the traditional Chinese medicine *Atractylodes macrocephala* promotes mitochondrial biogenesis by activating the PGC-1α/NRF1/TFAM axis, thereby alleviating exercise-induced fatigue [48]. Further research has shown that certain multi-active natural products, such as the soybean-derived compound marsdenia tenacissima extract B, can increase skeletal muscle mass and function by activating the SIRT1/PGC-1α pathway, providing new insights into the prevention and treatment of age-related sarcopenia [19]. These studies collectively reveal that bioactive components widely present in nature largely converge their mechanisms of action on the AMPK/SIRT1/PGC-1α signaling axis. By improving mitochondrial function and regulating protein metabolic homeostasis, they offer a wealth of candidate compounds and novel research perspectives for the intervention of metabolic muscle atrophy and age-related sarcopenia [45,46]. In summary, the screening of natural products targeting this signaling axis, along with mechanistic studies, holds promise for opening new avenues in the prevention and treatment of muscle atrophy-related diseases.

##### Neurogenic Atrophy

Neurogenic atrophy, with amyotrophic lateral sclerosis (ALS) as a representative condition, is fundamentally characterized by the progressive loss of motor neurons [49]. Skeletal muscle denervation resulting from this process is the direct cause of muscle atrophy; however, the underlying pathological mechanisms extend far beyond this, intricately involving mitochondrial dysfunction, oxidative stress, and neuroinflammation [4,50]. These factors mutually reinforce one another, establishing a vicious cycle that ultimately leads to irreversible loss of muscle function. Consequently, targeting key nodes within this network has emerged as a highly promising therapeutic strategy. Notably, these pathological processes are closely associated with dysregulation of the AMPK/SIRT1/PGC-1α signaling axis, a central hub governing energy metabolism.

In the classic SOD1^G93A^ mouse model of ALS, activating the AMPK/SIRT1/PGC-1α signaling axis has been demonstrated to possess significant therapeutic potential. Research indicates that the arctigenin derivative A-1 functions through a dual mechanism: on one hand, it activates the AMPK/SIRT1/PGC-1α pathway, promoting mitochondrial biogenesis and ameliorating the muscle energy metabolic deficits induced by motor neuron damage; on the other hand, it effectively suppresses neuroinflammation by modulating the AMPK/SIRT1/IL-1β/NF-κB pathway, thereby reducing motor neuron loss, alleviating muscle atrophy, and significantly improving motor function in model mice [37]. This suggests that simultaneously targeting mitochondrial function and inflammatory responses may represent an effective strategy for treating neurogenic atrophy such as ALS, with the AMPK/SIRT1/PGC-1α axis serving as the central hub for this combined therapeutic approach.

Furthermore, the classic anti-inflammatory drug aspirin has also been shown to exert protective effects against denervation-induced muscle atrophy in models of nerve injury. Aspirin has been shown to inhibit the transition from slow-twitch to fast-twitch muscle fibers by modulating the SIRT1/PGC-1α axis [51]. Similarly, celecoxib, a selective COX-2 inhibitor, has been shown to ameliorate muscle atrophy across multiple models, including denervation, hindlimb unloading, and diabetic sarcopenia, by suppressing inflammation, oxidative stress, and endoplasmic reticulum stress, while also improving microcirculation in denervated muscle [40,52,53]. These findings not only expand the pharmacological application of NSAIDs such as aspirin and celecoxib but also underscore the central role of regulating the SIRT1/PGC-1α axis and related inflammatory pathways in combating denervation-induced muscle atrophy, highlighting this pathway as a critical nexus linking inflammatory regulation and muscle fiber homeostasis.

In summary, whether through direct activation of the AMPK/SIRT1/PGC-1α pathway to restore mitochondrial function, or through modulation of its upstream or downstream nodes to suppress neuroinflammation and aberrant fiber-type switching, these studies collectively reveal the substantial potential of targeting the AMPK/SIRT1/PGC-1α pathway in the treatment of neurogenic atrophy. This pathway not only bridges energy metabolism and inflammatory responses but also acts as a “master switch” in maintaining motor neuron survival and muscle fiber homeostasis. In this context, beyond classical apoptosis and autophagy, ferroptosis—an iron-dependent lipid peroxidation-driven cell death implicated in neurological and muscular disorders—represents a novel mechanism through which the AMPK/SIRT1/PGC-1α axis, via its regulation of antioxidant defense, may protect muscle integrity [54,55]. Consequently, future studies targeting this pathway should consider ferroptosis as a potential downstream effector and therapeutic node in neurogenic atrophy.

#### 4.2.2. Muscular Dystrophy

Duchenne muscular dystrophy (DMD) is a severe, progressive muscle disorder caused by mutations in the dystrophin gene, characterized pathologically by persistent inflammation, myofiber necrosis, fatty infiltration, and fibrosis, ultimately culminating in loss of muscle function [56,57]. Notably, during the pathological progression of DMD, the activity of the AMPK/SIRT1/PGC-1α signaling axis is markedly suppressed, a key factor believed to contribute to disease exacerbation [56]. Given the central role of this pathway in regulating mitochondrial biogenesis, oxidative myofiber specification, and anti-inflammatory responses, restoring its activity represents a highly promising therapeutic strategy, making it a major focus in current DMD drug development efforts.

A substantial body of research confirms that targeted activation of the AMPK/SIRT1/PGC-1α pathway effectively ameliorates the pathological phenotype of DMD. For instance, the adipokine adiponectin, acting through its muscle-specific receptor AdipoR1, has been shown to activate the AMPK/SIRT1/PGC-1α signaling cascade. This not only promotes type I oxidative myofiber formation and upregulates utrophin to partially compensate for dystrophin deficiency but also significantly reduces muscle inflammation and damage, thereby demonstrating marked efficacy in improving muscle strength and alleviating pathology in DMD mouse models [56,58]. Resveratrol has also demonstrated protective effects in the mdx mouse model of DMD (see Section 5.2 for a detailed mechanistic discussion) [34]. Notably, while myostatin, a negative regulator of muscle growth, increases muscle mass upon genetic ablation, it may concurrently impair mitochondrial function by inhibiting the AMPK/SIRT1/PGC-1α pathway. This suggests that in DMD therapy, strategies focused solely on muscle hypertrophy without addressing mitochondrial health may be suboptimal [15]. Collectively, these findings establish the AMPK/SIRT1/PGC-1α pathway as a critical molecular target for the treatment of muscular dystrophy, providing a robust theoretical foundation for developing diverse therapeutic strategies.

Future research should prioritize the development of efficient and specific agents or interventions to activate this pathway, such as AdipoR agonists or SIRT1 activators, potentially in combination with gene therapy or cell-based approaches, to fundamentally improve muscle function and quality of life in DMD patients. Concurrently, in-depth investigations into the crosstalk between this pathway and other signaling networks, such as the NF-κB inflammatory pathway and the ubiquitin-proteasome degradation system, will provide the theoretical basis for devising more effective combination treatment strategies, ultimately facilitating a paradigm shift in DMD therapy from symptomatic management to disease modification.

#### 4.2.3. Insulin Resistance and Type 2 Diabetes Mellitus

Insulin resistance (IR) represents a core pathophysiological feature of type 2 diabetes mellitus (T2DM). Skeletal muscle, being the primary site for insulin-mediated glucose uptake, plays a pivotal role in maintaining systemic glucose homeostasis. Under insulin-resistant conditions, skeletal muscle exhibits impaired mitochondrial function, increased ectopic lipid accumulation, and elevated oxidative stress, alongside a marked downregulation of the AMPK/SIRT1/PGC-1α signaling pathway [59]. Consequently, activating this pathway to enhance mitochondrial function, ameliorate lipid metabolism, and alleviate oxidative stress is considered an effective approach for improving insulin sensitivity [4,10].

The mechanisms by which this pathway becomes dysregulated in insulin-resistant muscle are multifaceted. Reduced NAD^+^ levels impair SIRT1 activity, while chronic hyperglycemia and hyperlipidemia suppress AMPK phosphorylation, collectively diminishing PGC-1α-mediated mitochondrial biogenesis [9,60]. This metabolic deficit creates a vicious cycle: impaired mitochondrial function exacerbates lipid accumulation and oxidative stress, which further suppress AMPK/SIRT1/PGC-1α activity, perpetuating insulin resistance [4]. A diverse array of interventions, including exercise, pharmacological agents, and natural products, have been shown to ameliorate insulin resistance and diabetic myopathy by restoring activity of this axis [61,62,63]. Given that these interventions overlap substantially with those discussed for other muscle atrophy conditions (e.g., sarcopenia, cachexia), their detailed mechanisms, experimental models, and evidence levels are presented in Section 5.

In addition to skeletal muscle effects, activation of this pathway may also contribute to mitigating diabetic complications such as nephropathy and cardiomyopathy, which share common pathogenic drivers including metabolic dysregulation, oxidative stress, and fibrotic signaling. Thus, the AMPK/SIRT1/PGC-1α axis represents a promising therapeutic target not only for diabetic myopathy but also for broader diabetic complications. In summary, targeted activation of this pathway—whether through exercise, pharmacological agents, or natural products—offers a rational strategy for improving skeletal muscle metabolism and enhancing insulin sensitivity in T2DM.

## 5. Therapeutic Strategies Targeting the AMPK/SIRT1/PGC-1α Pathway

Given the central role of this pathway in maintaining skeletal muscle metabolic homeostasis, coupled with its significant dysregulation in various muscle disorders—such as sarcopenia, diabetic myopathy, and cachexia—targeting the AMPK/SIRT1/PGC-1α axis has emerged as a highly promising therapeutic strategy. Current research efforts are exploring effective methods to modulate this pathway from multiple dimensions, aiming to develop novel interventions that improve muscle function and delay disease progression. The evidence levels of these therapeutic strategies, categorized by experimental model, are summarized in Table 1.

### 5.1. Exercise and Physical Therapy

Exercise represents the most potent and natural physiological stimulus for activating the AMPK/SIRT1/PGC-1α axis. Numerous studies in rodent models and human subjects have demonstrated that both endurance and resistance exercise, by inducing metabolic energy stress, can activate this pathway to varying degrees, thereby conferring significant physiological benefits. For example, in aged mice, treadmill training activates the AMPK/SIRT1/PGC-1α pathway and ameliorates sarcopenia [64]. In humans, regular endurance exercise increases PGC-1α expression and SIRT1 activity in vastus lateralis muscle biopsies [65]. In C2C12 myotubes, mechanical stretch mimics exercise-induced AMPK phosphorylation [24]. These include promoting mitochondrial biogenesis, enhancing insulin sensitivity, increasing muscle endurance, and delaying age-related muscle decline [13,50,66,67]. The core mechanism involves an exercise-induced increase in the AMP/ATP ratio and in NAD^+^ levels, which activate AMPK and SIRT1, respectively. This, in turn, synergistically promotes the activation and deacetylation of PGC-1α, initiating a transcriptional program for downstream genes involved in mitochondrial biogenesis and oxidative metabolism [9,13]. Consequently, exercise constitutes the foundational strategy for improving skeletal muscle function and systemic metabolic health through this highly conserved energy-sensing pathway.

Notably, recent studies have further revealed broader protective mechanisms mediated by exercise through this pathway, indicating that its effects extend beyond local skeletal muscle to exert systemic regulatory functions. For instance, aerobic exercise not only acts directly on skeletal muscle but also upregulates kidney-derived ELABELA. This factor acts systemically on the heart, activating the APJ-AMPK-SIRT1 pathway in cardiac tissue and thereby ameliorating myocardial fibrosis following myocardial infarction [62]. Furthermore, exercise can upregulate the expression of irisin by modulating the miR-34a/SIRT1/PGC-1α/FNDC5 signaling axis, thereby improving diabetic cardiomyopathy [13]. These findings significantly enrich our understanding of the physiological effects of exercise, highlighting its capacity to synergistically maintain organismal homeostasis through inter-organ crosstalk [50]. Thus, the systemic benefits of exercise underscore its immense potential as an intervention to promote multi-system health.

Despite the notable efficacy of exercise therapy, its clinical application often faces challenges, including poor patient compliance and low feasibility for individuals who are severely ill or have mobility limitations. These constraints have spurred the emergence of the concept of “exercise mimetics”—pharmaceuticals or nutrients that replicate the molecular effects of exercise, offering an alternative or complementary strategy for patients unable to engage in adequate physical activity [50,68]. An ideal exercise mimetic would selectively activate key signaling pathways such as AMPK/SIRT1/PGC-1α, thereby recapitulating many of the benefits of exercise without the need for high-intensity physical exertion. Therefore, developing efficient and safe exercise mimetics holds significant promise for expanding the clinical applicability of exercise-based therapeutic approaches.

### 5.2. Nutrition and Natural Products

A substantial body of research has confirmed that a variety of nutrients and natural products exert beneficial effects in maintaining skeletal muscle homeostasis and ameliorating muscle dysfunction by activating the AMPK/SIRT1/PGC-1α signaling axis. These naturally derived compounds provide a rich source of candidate molecules for developing safe and effective interventions for sarcopenia. Notably, this signaling axis not only responds to exogenous compounds but also mediates the beneficial effects of physiological interventions such as exercise and caloric restriction [17,33]. Consequently, natural products can function as “exercise mimetics” or “caloric restriction mimetics” [68]. These findings establish a solid theoretical foundation for identifying active molecules from natural products that mimic physiological interventions.

Polyphenolic compounds represent one of the most extensively studied categories of natural products, with resveratrol being a classic natural activator of SIRT1. In C2C12 myotubes, resveratrol promotes fast-to-slow fiber type conversion via the AMPK/SIRT1/PGC-1α pathway [22]. In aged rats, oral resveratrol supplementation (50 mg/kg/day for 8 weeks) activates SIRT1 and improves muscle mass and grip strength [7]. However, in a clinical trial of elderly subjects (NCT02514382), resveratrol alone failed to significantly improve muscle function, highlighting the translational gap [7]. Furthermore, the combination of resveratrol and metformin synergistically enhances myogenesis by increasing irisin release [24]. Beyond resveratrol, other polyphenols have also demonstrated significant potential. For instance, luteolin and pterostilbene can ameliorate high-fat diet-induced obesity and associated muscle dysfunction by activating this pathway, with luteolin additionally shown to improve mitochondrial quality control [46,69]. The combination of curcumin with exercise training synergistically increases cAMP levels in muscle, thereby more effectively promoting mitochondrial biogenesis via activation of the PKA/CREB/LKB1 pathway [70]. Notably, research on other polyphenolic components such as vitexin is also contributing new evidence to this field. In summary, polyphenolic compounds exhibit broad application prospects in maintaining muscle function through multi-target, multi-pathway synergistic effects.

Saponins, as another class of natural products with significant biological activity, also play a key role in regulating this signaling pathway. Studies have confirmed that soyasapogenol B promotes mitochondrial biogenesis by activating the SIRT1/PGC-1α pathway, thereby ameliorating sarcopenia; molecular docking studies suggest it may directly bind to SIRT1, offering new insights for developing direct SIRT1 activators [19]. Additionally, *Panax japonicus* saponins have been found to inhibit cardiomyocyte apoptosis by modulating the AMPK/SIRT1/NF-κB signaling pathway in aging models, providing a new perspective for ameliorating age-related systemic functional decline, including skeletal muscle function [71]. These studies reveal the unique value of saponins as direct or indirect SIRT1 modulators in intervening in aging-related muscle dysfunction.

Beyond these classic compound categories, complex extracts derived from plants or traditional herbs, as well as novel nanovesicles, also show considerable potential for regulating muscle metabolism via this pathway. For example, goji berry-derived nanovesicles (GqDNVs) effectively ameliorate dexamethasone-induced muscle atrophy by activating the AMPK/SIRT1/PGC-1α pathway, and metabolomic analysis has revealed their regulatory effects on amino sugar, nucleotide sugar metabolism, and oxidative phosphorylation processes [35]. Chives-derived extracellular vesicles (CL-EVs) exhibit a dual regulatory mechanism, promoting mitochondrial biogenesis by activating the AMPK/SIRT1/PGC-1α pathway while simultaneously inhibiting the Akt/FoxO3a/Atrogin-1/MuRF1 protein degradation pathway, thereby synergistically improving sarcopenia. This “multi-target and gut-muscle axis” regulatory model offers an innovative strategy for treating sarcopenia [36]. Paeoniflorin has also been reported to alleviate CKD-associated muscle atrophy via this pathway (see Section 4.2.1 for a detailed discussion) [18]. Salidroside exerts protective effects against diabetic nephropathy through SIRT1/PGC-1α axis-mediated mitochondrial biogenesis, suggesting its potential in ameliorating systemic metabolic disorders [72]. Collectively, these novel natural products and their derived formulations provide diversified intervention strategies for muscle atrophy in the context of complex diseases by targeting the AMPK/SIRT1/PGC-1α axis.

Essential nutrients also play an indispensable role in regulating this signaling axis. Research reveals that the combination of leucine and metformin synergistically activates SIRT1 even under conditions of low intracellular NAD^+^ levels, subsequently improving insulin sensitivity through the AMPK pathway and extending lifespan in *C. elegans* models, demonstrating the synergistic potential of nutrient-drug combinations [11]. ω-3 polyunsaturated fatty acids, particularly eicosapentaenoic acid (EPA) found in fish oil, have also been shown to ameliorate type 2 diabetes-related sarcopenia by activating the AMPK/SIRT1/PGC-1α pathway, with mechanisms involving improved insulin resistance, reduced accumulation of advanced glycation end products (AGEs), and suppression of oxidative stress and inflammation [26,73]. Additionally, alpha-lipoic acid has been found to effectively preserve skeletal muscle mass in a type 2 diabetic rat model by simultaneously activating the AMPK/SIRT1/PGC-1α and AKT/mTOR/p70S6K pathways [74]. Vitamin D has also been confirmed to alleviate palmitate-induced lipid accumulation and mitochondrial dysfunction in C2C12 myotubes by activating the AMPK/SIRT1 signaling pathway [75]. These findings underscore the central role of essential nutrients in maintaining muscle energy metabolic homeostasis and provide a scientific basis for developing nutrient-based synergistic intervention strategies.

Although the aforementioned natural products offer abundant candidate molecules for treating muscle atrophy, their clinical application still faces numerous challenges. Key issues include low oral bioavailability (e.g., resveratrol undergoes rapid glucuronidation and sulfation), metabolic instability, and poorly defined dose–response relationships in humans. Moreover, long-term safety profiles remain largely unknown for many compounds. For instance, studies in mdx mice suggest that excessively high doses or prolonged treatment durations with resveratrol may fail to sustain pathway activation or even produce suboptimal effects, underscoring the importance of dose optimization [34]. Therefore, future research should focus on improving in vivo efficacy through structural modification, exploring rational combination strategies (e.g., with exercise or other drugs), and utilizing novel delivery systems such as plant-derived exosomes [36,76]. Only by systematically addressing these translational medicine challenges can promising natural products ultimately advance from the laboratory to the clinic.

### 5.3. Small Molecule Drugs and Targeted Compounds

Given the central role of the AMPK/SIRT1/PGC-1α signaling axis in maintaining skeletal muscle metabolic homeostasis, drug development targeting key molecules within this pathway has emerged as a focal point in translational medicine research. The core objective is to mimic the health benefits of exercise or caloric restriction through precise pharmacological intervention, thereby providing novel strategies for treating conditions such as muscle atrophy and metabolic diseases. Consequently, exploring and developing drug molecules that safely and effectively modulate this pathway holds significant clinical translational value.

#### 5.3.1. AMPK Activators

As a central sensor of energy metabolism, AMPK represents a primary target for pharmacological intervention. Metformin, a classic AMPK activator, is well-established for its glucose-lowering effects in diabetic patients. Moreover, recent studies have revealed its novel function in promoting myogenesis via the AMPK/SIRT1/PGC-1α pathway in C2C12 cells and high-fat diet-induced obese mice [24]. However, in a small pilot study of non-diabetic older adults (*n* = 20, 12 weeks of metformin), no significant improvement in muscle mass was observed, suggesting that metformin’s effects may be context-dependent [24]. Specifically, metformin enhances the release of irisin from muscle cells by activating the AMPK/SIRT1/PGC-1α pathway, thereby improving muscle function and offering a new perspective for the treatment of muscle atrophy [24]. Additionally, AICAR, a widely used direct AMPK agonist, effectively mimics certain effects of exercise in cellular and animal models, such as enhancing mitochondrial function and inducing slow-twitch muscle fiber conversion [15,29]. Notably, numerous natural products have also been validated as effective activators of this pathway. For instance, berberine has been shown to activate this pathway in aging models (see Section 4.2.1 for a detailed discussion) [31]; meanwhile, Alpha-lipoic acid effectively preserves skeletal muscle mass in a type 2 diabetic rat model by upregulating the AMPK/SIRT1/PGC-1α signaling pathway [74]. Similarly, other natural compounds such as paeoniflorin and ampelopsin [29] have been shown to alleviate skeletal muscle atrophy through activation of this pathway (see Section 4.2.1 for paeoniflorin) [18,28]. In summary, through their diverse molecular structures, AMPK activators collectively demonstrate significant potential in mimicking the benefits of exercise and counteracting muscle atrophy.

#### 5.3.2. SIRT1 Activators

As a deacetylase, SIRT1 serves as a critical link connecting energy metabolism and mitochondrial function. Resveratrol, a well-studied natural SIRT1 activator, promotes fast-to-slow muscle fiber conversion via the AMPK/SIRT1/PGC-1α pathway, though its effects are dose dependent with excessive doses potentially failing to sustain activation (see Section 5.2 for a comprehensive discussion) [22,34]. Beyond resveratrol, synthetic SIRT1 activators such as the small molecule SRT1720 can mimic certain effects of caloric restriction in animal models, significantly improving mitochondrial function and metabolic health [9]. Furthermore, several plant extracts demonstrate potent SIRT1-activating capabilities. For example, Salidroside improves mitochondrial function in diabetic nephropathy mice via the SIRT1/PGC-1α axis [72]. Moreover, Stevia extract has been found to alleviate muscle atrophy under diabetic conditions by upregulating SIRT1/PGC-1α signaling [77]. Recent research also identified that Soyasapogenol B increases muscle mass and function by activating the SIRT1/PGC-1α pathway, providing a novel strategy for preventing sarcopenia [19]. Collectively, these findings suggest that targeting SIRT1 and its downstream pathways represents an effective strategy for improving muscle energy metabolism and function.

#### 5.3.3. Other Targeted Drugs and Multi-Target Strategies

Beyond compounds directly acting on AMPK or SIRT1, novel applications for drugs traditionally indicated for other conditions have been discovered via this pathway, fully illustrating the potential of drug repurposing. For instance, Tadalafil, a phosphodiesterase type 5 inhibitor used for erectile dysfunction, was found to improve mitochondrial dysfunction in diabetic cardiomyopathy by enhancing NO signaling and activating the SIRT1/PGC-1α pathway [78]. Similarly, the anti-inflammatory drug Colchicine was shown to inhibit NLRP3 inflammasome-mediated cardiomyocyte pyroptosis by activating the AMPK/SIRT1 signaling pathway, offering a new strategy for treating myocardial injury associated with coronary microembolization [79]. Additionally, the angiotensin II receptor antagonist Telmisartan improves insulin sensitivity by activating the AMPK/SIRT1 pathway in muscle, an effect independent of PPARγ [80]. Of further interest, the GLP-1 receptor agonist Liraglutide has been found to reduce tenocyte inflammation and endoplasmic reticulum stress by activating the GLP-1R-AMPK/SIRT1 pathway, thereby promoting rotator cuff tear repair [63]. These discoveries collectively underscore the significant potential of this signaling pathway as a convergence point for diverse pharmacological interventions, laying a solid foundation for developing “one stone, multiple birds” drug strategies aimed at complex metabolic diseases.

### 5.4. Gene and Cellular Therapies

Compared with nutritional and pharmacological interventions, achieving regulation at the more upstream levels of gene and cellular control offers a more promising strategy for achieving durable and efficient treatment of muscle atrophy. These approaches aim to correct or compensate for the key defects underlying muscle metabolic imbalance at their root, potentially enabling more fundamental therapeutic outcomes.

#### 5.4.1. Genetic Regulation

Through gene overexpression or knockout techniques, researchers are intensively exploring the regulatory roles of key genes within this pathway and validating their potential as therapeutic targets. For instance, in a Drosophila model, muscle-specific overexpression of the autophagy-related gene *Atg2* effectively delays aging in skeletal muscle and heart and extends lifespan by activating the AMPK/SIRT1/PGC-1α pathway [81]. In mouse models, however, constitutive activation of this pathway via genetic manipulation has shown mixed results, and no human studies have been reported to date. Therefore, gene therapy targeting this axis remains at an early, preclinical stage. Furthermore, muscle-specific overexpression of the *Presenilin* (*Psn*) gene has also been shown to delay muscle aging by modulating the homeostasis of the SIRT1/PGC-1α pathway [82]. However, the complexity of genetic manipulation necessitates careful assessment of its potential risks. For example, although *Myostatin* gene knockout significantly increases muscle mass, it leads to mitochondrial dysfunction, with one of the underlying mechanisms being the inhibition of AMPK/SIRT1/PGC-1α pathway activity [15]. This finding highlights the importance of precise regulation in therapeutic interventions, avoiding the induction of metabolic imbalance and mitochondrial dysfunction resulting from excessive promotion of muscle growth. Consequently, future gene therapy strategies may need to focus not only on the overexpression or inhibition of single genes but also on exploring how to finely modulate multiple nodes within the network to achieve a balance between therapeutic efficacy and metabolic homeostasis.

#### 5.4.2. Cell Therapies and Novel Delivery Systems

Utilizing extracellular vesicles (EVs) as natural or engineered delivery vehicles to target the AMPK/SIRT1/PGC-1α pathway represents a highly promising therapeutic strategy. EVs mediate intercellular communication by delivering bioactive molecules (such as proteins, nucleic acids, and lipids) to target cells and offer advantages including low immunogenicity, high biocompatibility, and the potential for engineering modifications.

Of particular note are plant-derived nanovesicles, which have garnered significant attention due to their low immunogenicity, high yield, and potential for cross-kingdom molecular delivery. These vesicles are rich in lipids, sugars, and various bioactive molecules, and their mechanisms of action are gradually being elucidated. For instance, nanovesicles derived from *Lycium barbarum* (GqDNVs) and extracellular vesicles derived from chive leaves (CL-EVs) have been shown to ameliorate muscle atrophy by activating this pathway (see Section 5.2 for a detailed discussion) [35,36]. These studies not only validate the feasibility of EVs as “cell-free” therapeutic carriers but, more importantly, open up a novel avenue for applying the traditional Chinese medicine concept of “medicinal and food homology” in modern therapy. By delivering the active components of natural products in the form of nanoscale vesicles, this strategy may achieve more efficient targeting and therapeutic effects.

**Figure 4:**
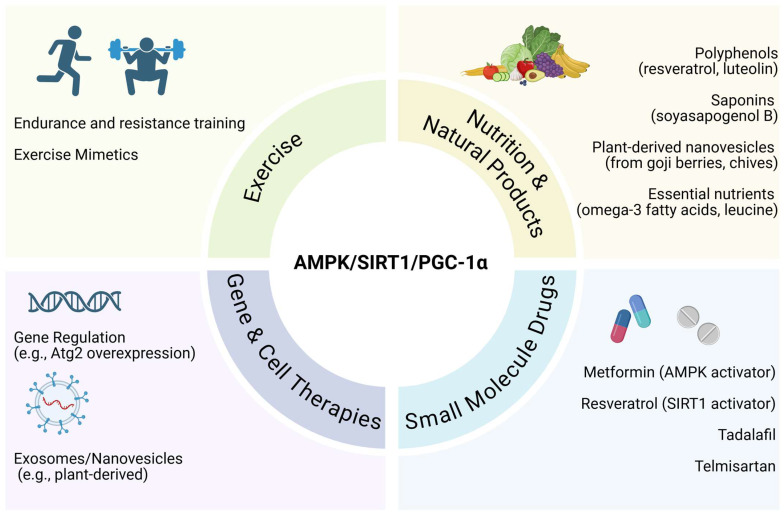
Therapeutic strategies targeting the AMPK/SIRT1/PGC-1α axis. The figure categorizes interventions into four groups: (1) Nutrition and natural products—polyphenols (resveratrol, luteolin), saponins (soyasapogenol B), plant-derived nanovesicles (from goji berries, chives), and essential nutrients (omega-3 fatty acids, leucine); (2) Small molecule drugs—metformin, resveratrol, tadalafil, and telmisartan; (3) Gene regulation—e.g., *Atg2* overexpression; (4) Cell-based therapies and delivery systems—exosomes and nanovesicles (e.g., plant-derived). All converge on the central axis to promote mitochondrial biogenesis, metabolic homeostasis, and muscle function. Abbreviations: AMPK, AMP-activated protein kinase; SIRT1, sirtuin 1; PGC-1α, peroxisome proliferator-activated receptor gamma coactivator 1-alpha; Atg2, autophagy-related protein 2. Created in BioRender. Jie, W. (2026) https://BioRender.com/vqw4mz4.

In summary, strategies targeting the AMPK/SIRT1/PGC-1α signaling axis are becoming increasingly refined and comprehensive, ranging from active ingredients in natural products to small-molecule drugs targeting key pathway nodes and further to precision interventions at the genetic and cellular levels (Figure 4). Collectively, these studies paint a blueprint for multi-layered, multi-targeted therapeutics. Future research will focus on addressing core issues such as bioavailability, safety, specificity, and long-term efficacy of these interventions, with the ultimate goal of translating these foundational research findings into novel clinical strategies capable of effectively combating muscle atrophy and related metabolic diseases.

## 6. Challenges and Future Perspectives

**Figure 5:**
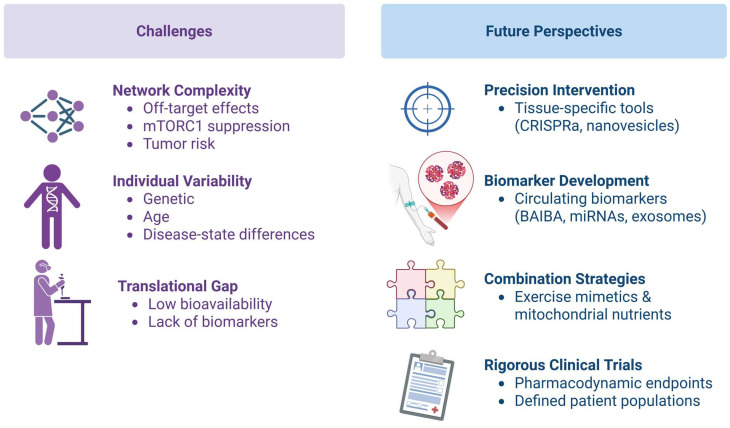
Challenges and future perspectives for clinical translation of AMPK/SIRT1/PGC-1α-targeted therapies. Left panel (Challenges): Major obstacles include (1) signaling network complexity and off-target effects (mTORC1 suppression, tumorigenesis risk); (2) individual variability (genetic background, age, disease state); (3) translational gap (poor bioavailability, lack of robust pharmacodynamic biomarkers). Right panel (Future Perspectives): Proposed solutions include (1) precision intervention tools (tissue-specific CRISPRa, targeted nanovesicles); (2) biomarker development (circulating BAIBA, miRNAs, exosomal content); (3) combination strategies (exercise mimetics with mitochondrial nutrients); (4) rigorous clinical trials with pharmacodynamic endpoints and well-defined patient populations. Abbreviations: AMPK, AMP-activated protein kinase; SIRT1, sirtuin 1; PGC-1α, peroxisome proliferator-activated receptor gamma coactivator 1-alpha; mTORC1, mechanistic target of rapamycin complex 1; CRISPRa, clustered regularly interspaced short palindromic repeats activation; BAIBA, β-aminoisobutyric acid; miRNAs, microRNAs. Created in BioRender. Jie, W. (2026) https://BioRender.com/zun3zti.

Although the AMPK/SIRT1/PGC-1α axis holds significant promise as a therapeutic target for skeletal muscle-related disorders, its clinical translation faces several formidable challenges. Addressing the core issue of how to translate these fundamental insights into safe and effective patient applications is imperative for the field (Figure 5). Only by systematically tackling these challenges can the promise of basic research be transformed into effective therapies that benefit patients.

### 6.1. Complexity of the Signaling Network and Off-Target Effects

As critical metabolic sensors and transcriptional coactivators within the cell, AMPK, SIRT1, and PGC-1α are involved in regulating a broad spectrum of physiological processes far beyond mitochondrial biogenesis and energy metabolism, effectively functioning as cellular “multitaskers.” Consequently, systemic activation of these molecules carries a high risk of eliciting widespread off-target effects and potential adverse events.

AMPK overactivation and anabolic suppression. While moderate AMPK activation promotes catabolic processes to restore energy balance, excessive or chronic activation may over-suppress anabolic pathways centered on mTORC1. Given that mTORC1 is essential for protein synthesis and muscle hypertrophy, its sustained inhibition could potentially hinder muscle mass maintenance or even exacerbate atrophy under certain conditions [24,28]. This delicate balance suggests a narrow therapeutic window for AMPK-targeted interventions.

SIRT1 hyperactivation and oncogenic risks. SIRT1 is highly expressed in various tumors, and its hyperactivation in specific pathological contexts may promote tumor growth and chemoresistance by inhibiting apoptosis and facilitating metabolic reprogramming [83]. Therefore, indiscriminate systemic activation of SIRT1—even for the purpose of treating muscle atrophy—raises safety concerns, particularly in aging populations where cancer risk is already elevated.

Need for tissue-specific interventions. These off-target risks underscore the urgent need for tissue-specific, preferably skeletal muscle-specific, targeting. Strategies such as nanomaterial-based delivery (e.g., plant-derived nanovesicles [36,84]) or gene therapy driven by muscle-specific promoters represent crucial directions for achieving precise modulation and avoiding systemic side effects. Alternatively, exploring strategies that selectively activate specific downstream effectors within skeletal muscle may open new avenues for precision regulation.

Crosstalk with immune and inflammatory pathways. Beyond the classical metabolic networks, the AMPK/SIRT1/PGC-1α axis also exhibits extensive crosstalk with immune signaling cascades. Recent studies have identified the cGAS-STING pathway as a central regulator of sterile inflammation and muscle atrophy [2]. Notably, AMPK can suppress cGAS-STING signaling by limiting mitochondrial DNA release, while SIRT1 deacetylates and inhibits STING. Conversely, chronic AMPK/SIRT1 downregulation in aging or disease may derepress the cGAS-STING pathway, exacerbating inflammatory muscle damage. Furthermore, macrophage polarization—a key determinant of muscle regeneration versus fibrosis—is also modulated by this axis. AMPK/SIRT1 activation promotes an M2 (anti-inflammatory, pro-regenerative) macrophage phenotype, whereas pathway suppression favors M1 (pro-inflammatory) polarization, which aggravates atrophy [85,86]. These intricate interactions between metabolic and immune networks highlight that targeting the AMPK/SIRT1/PGC-1α axis in isolation may be insufficient; combination strategies that concurrently modulate inflammatory pathways may be required for optimal therapeutic outcomes.

In addition to the aforementioned off-target risks, the AMPK/SIRT1/PGC-1α axis exhibits intricate crosstalk with immune and stress-response pathways. Recent studies have identified the cGAS-STING pathway as a central regulator of sterile inflammation and muscle atrophy. Notably, AMPK can suppress cGAS-STING signaling by limiting mitochondrial DNA release, while SIRT1 deacetylates and inhibits STING. Conversely, chronic downregulation of AMPK/SIRT1 in aging or disease may derepress the cGAS-STING pathway, exacerbating inflammatory muscle damage. Furthermore, macrophage polarization—a key determinant of muscle regeneration versus fibrosis—is also modulated by this axis. AMPK/SIRT1 activation promotes an M2 (anti-inflammatory, pro-regenerative) macrophage phenotype, whereas pathway suppression favors M1 (pro-inflammatory) polarization, which aggravates atrophy. These interconnections between metabolic and immune networks highlight that targeting the AMPK/SIRT1/PGC-1α axis in isolation may be insufficient; combination strategies that concurrently modulate inflammatory pathways may be required for optimal therapeutic outcomes. This complexity is also reflected in the challenges presented in Figure 5.

### 6.2. Individual Variability and the Therapeutic Window

Responses to a given intervention can vary drastically among individuals due to factors such as age, sex, genetic background, disease status, and comorbidities. This inter-individual heterogeneity poses a major obstacle to clinical implementation. For example, although the activity of the AMPK/SIRT1/PGC-1α axis generally declines with age, the rate of decline and residual function can differ substantially between individuals, influenced by genetic polymorphisms (e.g., LGR4 [87]), lifestyle, and metabolic status. Future therapeutic strategies must therefore move beyond a “one-size-fits-all” model toward individualized approaches grounded in precise assessment. This necessitates the development of convenient, dynamic biomarkers that reflect pathway activity in skeletal muscle (such as specific circulating metabolites, microRNAs, or exosomal contents) to determine the optimal timing and combination of interventions. Moreover, the timing of the “therapeutic window” is paramount. In patients with advanced disease and severe muscle atrophy, simply activating mitochondrial biogenesis may be insufficient to reverse established structural and functional damage, likely requiring multifaceted strategies that combine anti-inflammatory, anti-apoptotic, and pro-regenerative approaches [4]. Hence, future research should focus on creating diagnostic tools capable of accurately identifying distinct pathological stages and molecular subtypes in patients, thereby laying the foundation for tailored, individualized therapies.

### 6.3. The Gap Between Basic Research and Clinical Translation

Numerous natural products or compounds that demonstrate robust efficacy in cellular and animal models often yield disappointing results, or even fail outright, upon entering clinical trials. This translational gap between basic research and clinical application primarily stems from the following factors: (1) Low bioavailability and metabolic instability: Many active components (e.g., resveratrol) suffer from poor oral absorption and rapid metabolic inactivation in vivo, making it difficult to achieve effective concentrations in target tissues; (2) Inappropriate administration routes and dosages: Preclinical studies frequently employ administration routes not commonly used in clinical practice, such as intraperitoneal injection, or utilize dosages far exceeding those clinically feasible; (3) Lack of robust pharmacodynamic biomarkers: Clinical trial designs often fail to effectively verify whether the drug successfully engages its intended target in patients (i.e., whether the pathway is activated). Therefore, future clinical trial designs must be more rigorous. We strongly recommend incorporating the assessment of surrogate endpoints, such as directly measuring the phosphorylation levels, acetylation status, and expression of downstream target genes of key pathway proteins through muscle biopsies [7]. Concurrently, integrating comprehensive pharmacokinetic/pharmacodynamic studies to clarify the exposure-response relationship in humans is an essential step to bridge this translational divide. Furthermore, employing preclinical models that more closely recapitulate the complexity of human diseases, alongside the early introduction of biomarker-guided dose-finding strategies, will help screen the most promising drug candidates before clinical trials are initiated, thereby significantly enhancing translational success rates.

### 6.4. Potential and Challenges of Combination Intervention Strategies

Given the complexity of skeletal muscle diseases, which involve imbalances across multiple processes such as protein synthesis and degradation, mitochondrial function, inflammation, and oxidative stress, interventions targeting a single pathway often fail to achieve optimal therapeutic outcomes. Consequently, “multi-target” and “combination intervention” strategies that target multiple pathways or multiple nodes within the same pathway are garnering increasing attention [7,24,88]. The concept of synergistic efficacy has been validated in several studies. For instance, combined exercise and resveratrol intervention demonstrated synergistic effects in ameliorating age-related muscle dysfunction [7]; co-administration of metformin and resveratrol synergistically enhanced myogenesis and promoted irisin release via activation of the AMPK/SIRT1/PGC-1α pathway [24]; and the combined application of electroacupuncture and sulforaphane more effectively repaired mitochondrial damage to improve sarcopenia by synergistically activating this pathway [88]. Future research should systematically explore the synergistic mechanisms between “exercise mimetics” (e.g., AMPK activators, AdipoR agonists [68]) and “mitochondrial nutrients” (e.g., omega-3 fatty acids, resveratrol). Additionally, Traditional Chinese Medicine (TCM) formulas, with their holistic regulatory advantages characterized by “multi-components and multi-targets,” may naturally align with the concept of combination intervention. Their potential in activating the AMPK/SIRT1/PGC-1α pathway to treat muscle atrophy, myocardial injury, and other conditions warrants in-depth investigation.

### 6.5. Perspectives and Future Directions

Based on current research progress and the challenges that remain, it is my view that future investigations in this field should focus on the following frontier directions to facilitate the substantive translation of fundamental research into clinical applications. Among these directions, three unresolved questions stand out as particularly critical for advancing the field: the risks of chronic AMPK activation, the definition of an optimal therapeutic window, and the need to balance mitochondrial biogenesis with quality control.

#### 6.5.1. Deepening the Study of Spatiotemporal and Cellular Heterogeneity in Mechanisms

In addition to further elucidating the crosstalk between the AMPK/SIRT1/PGC-1α pathway and pathways such as mTOR, NF-κB, endoplasmic reticulum stress, and autophagy under specific pathological conditions, future research should pay greater attention to its regulatory differences across distinct skeletal muscle fiber types (fast-twitch vs. slow-twitch) [23,89]. Notably, variations in the responsiveness of different muscle fibers to this signaling axis may be key to achieving precision intervention. Therefore, an in-depth exploration of the molecular mechanisms underlying these differences will facilitate the development of precise intervention strategies targeting specific muscle groups or functions, thereby enabling individualized regulation of muscle function.

In addition to exploring fiber-type-specific responses, several fundamental unresolved questions warrant dedicated investigation. (1) Does chronic AMPK activation lead to excessive catabolism? While acute AMPK activation during exercise is beneficial, prolonged pharmacological activation may sustain FOXO3a-mediated upregulation of Atrogin-1 and MuRF1, potentially tipping the balance toward net protein loss. Future studies should define the duration and magnitude of AMPK activation that maximizes mitochondrial benefits without compromising muscle mass. (2) What is the optimal therapeutic window for pathway activation? The response to AMPK/SIRT1/PGC-1α targeting is likely biphasic and context-dependent. For example, in early sarcopenia, moderate activation may restore homeostasis, whereas in advanced cachexia with severe mitochondrial dysfunction, more aggressive or combinatorial approaches may be needed. Biomarker-guided stratification will be essential to identify patients most likely to benefit. (3) How can we balance biogenesis and quality control? An exclusive focus on promoting mitochondrial biogenesis (e.g., via PGC-1α overexpression) without parallel enhancement of mitophagy may increase the burden of dysfunctional mitochondria, creating a “biogenesis without clearance” paradox. Preclinical studies suggest that simultaneous activation of both PGC-1α and mitophagy regulators (e.g., ULK1, Parkin) yields superior outcomes. Addressing these questions will require well-designed longitudinal studies in animal models and, eventually, in humans.

#### 6.5.2. Developing Precise and Controllable Intervention Tools

Leveraging gene-editing technologies (such as CRISPRa) or nucleic acid aptamers to develop tools that specifically enhance the activity or expression of PGC-1α or its upstream regulators (such as LGR4 [87]) in skeletal muscle represents a crucial strategy for avoiding systemic side effects. Concurrently, utilizing circulating exosomes or plant-derived nanovesicles [36] as “intercellular messengers” to mimic the systemic effects of exercise offers a highly promising alternative for populations unable to engage in conventional physical activity. This strategy, combining “precision targeting” with “systemic mimicking,” holds the potential to yield a new class of highly efficient and safe biologic agents.

#### 6.5.3. Establishing and Validating Effective Biomarker Systems

Identifying and validating circulating biomarkers that reliably reflect the activity of the AMPK/SIRT1/PGC-1α pathway in skeletal muscle is of paramount importance. Beyond traditional metabolites, focused attention should be directed towards novel markers such as specific microRNAs (e.g., miR-22-3p, miR-30d-5p) [23,66], and the protein and nucleic acid content of exosomes. The development and application of these markers will significantly advance the formulation of personalized treatment plans and the precise evaluation of clinical trials, providing critical monitoring tools for clinical translation.

#### 6.5.4. Designing Rigorous and Pragmatic Clinical Trials with Pharmacodynamic Biomarkers

Building upon robust mechanistic research and preclinical animal data, it is essential to design well-controlled clinical trials that validate the efficacy and safety of the most promising candidate agents (particularly naturally derived compounds and repurposed drugs with favorable safety profiles, such as metformin, resveratrol, and aspirin) in specific patient populations (e.g., sarcopenia, diabetic myopathy, cachexia). A critical but often overlooked component is the incorporation of reliable pharmacodynamic biomarkers that directly confirm target engagement in skeletal muscle. For example, measuring phosphorylation levels of AMPK, acetylation status of PGC-1α, or expression of downstream target genes (such as NRF1, TFAM) in muscle biopsies—or their circulating surrogates (e.g., specific microRNAs, β-aminoisobutyric acid, exosomal content)—would provide evidence that the intervention indeed activates the intended pathway. Such biomarker-guided dose-finding and proof-of-mechanism studies are essential to bridge the translational gap. Concurrently, given the established benefits of exercise, there should be active exploration of optimal “exercise-drug” combination strategies to provide effective alternative or complementary therapies for individuals unable to engage in regular physical activity (such as critically ill patients or the very elderly). Only through high-quality clinical evidence incorporating robust pharmacodynamic endpoints can laboratory discoveries ultimately be translated into effective treatments that benefit patients. Furthermore, given the emerging recognition that promoting mitochondrial biogenesis without enhancing mitophagy may be counterproductive, future clinical trials should explore combination strategies that simultaneously target both processes—for example, pairing an AMPK/PGC-1α activator (e.g., exercise or metformin) with a mitophagy enhancer (e.g., urolithin A or spermidine). Such dual-pronged approaches may more effectively restore mitochondrial homeostasis and achieve durable improvements in muscle mass and function.

#### 6.5.5. Exploring Novel Regulatory Nodes

Beyond the classical pathways involving AMPK and SIRT1, we should actively broaden our perspective to investigate new upstream regulators of this pathway. For instance, the discoveries of the G protein-coupled receptor LGR4 [87], and non-coding RNAs (such as miR-22-3p, miR-30d-5p) [23,66] provide us with novel targets for drug development. Furthermore, myokines such as irisin [24] and IL-15 [90], acting as downstream effector molecules and potential paracrine/endocrine signals of this pathway, may themselves represent entry points for therapeutic intervention. In-depth investigation of these novel regulatory nodes promises to open up entirely new avenues for the study of skeletal muscle metabolic regulation and disease treatment, offering more possibilities for addressing refractory muscle disorders.

## 7. Summary

In summary, the AMPK/SIRT1/PGC-1α signaling axis serves as a central hub in the regulation of skeletal muscle metabolism, orchestrating mitochondrial biogenesis, muscle fiber type switching, and protein homeostasis through the integration of diverse signals, including those related to energy and redox balance. Dysregulation of this pathway constitutes a common core molecular mechanism underlying the pathogenesis of various skeletal muscle disorders, such as sarcopenia, disuse muscle atrophy, and diabetic myopathy. Therapeutic strategies targeting this pathway have shown considerable promise, encompassing a wide range of approaches from physiological exercise to pharmacological agents, natural products, and gene-based interventions. However, the clinical translation of these findings faces critical challenges, including the complexity of the signaling network, individual variability, and issues of bioavailability. Accordingly, future research should focus on: (1) developing tissue-specific precision intervention tools to mitigate off-target effects; (2) establishing robust biomarker systems that reflect target activity to guide personalized therapy; and (3) exploring the synergistic effects of combination strategies, such as “exercise mimetics” and “mitochondrial nutrients.” By systematically addressing these key issues in translational medicine, it is anticipated that insights from fundamental research can be effectively translated into novel clinical solutions that benefit patients.

## Figures and Tables

**Table 1:** Therapeutic interventions targeting the AMPK/SIRT1/PGC-1α axis: evidence level, model systems, and translational limitations.

Intervention Category	Specific Examples	Model Systems (In Vitro/In Vivo)	Human Evidence for Muscle Atrophy	Evidence Level *	Key Limitations for Translation
Exercise and physical therapy	Endurance, resistance exercise	C2C12, primary myotubes; rodents (rats, mice), zebrafish	Limited RCTs in elderly/diabetic	I/II/III	Compliance, feasibility in frail patients
Polyphenols	Resveratrol, luteolin, curcumin, pterostilbene	C2C12; aged rats, mdx mice, HFD mice	Pilot studies only; no confirmatory RCTs	I/II/III	Low bioavailability, rapid metabolism, unclear optimal dose
Saponins	Soyasapogenol B, *Panax japonicus* saponins	C2C12; aged rats, CKD rats	None reported	II/III	Poor absorption, lack of human PK/PD data
Plant-derived nanovesicles and EVs	GqDNVs (goji berry), CL-EVs (chives), engineered exosomes	C2C12; dexamethasone-induced and aged mice	None reported	II/III	Scalability, storage stability, manufacturing complexity, unknown safety profile
Essential nutrients	Omega-3, leucine, vitamin D, ALA	C2C12; T2DM rats, pigs, HFD mice	Observational studies; small trials	I/II/III	Weak effect size, likely need combination therapy
AMPK activators	Metformin, AICAR, berberine, paeoniflorin	C2C12, L6 myotubes; aged rats, diabetic models, CKD models	Metformin: RCTs for T2DM (not for atrophy); others: none	II/III (for atrophy)	Off-target effects, poor specificity for muscle
SIRT1 activators	Resveratrol, SRT1720, salidroside	C2C12; mdx mice, diabetic nephropathy mice	RCTs conducted for resveratrol, but results inconsistent/negative for atrophy	I/II/III	Poor bioavailability; synthetic activators have toxicity concerns
Repurposed drugs	Tadalafil, telmisartan, liraglutide, colchicine, aspirin, celecoxib	Various cell lines; rats/mice—denervation atrophy, diabetic cardiomyopathy, coronary microembolization	RCTs exist for original indications; none for muscle atrophy	II/III (for atrophy)	Preclinical evidence only; off-target effects
Gene therapy	Atg2/Psn overexpression; myostatin knockout	C2C12 (mammalian); Drosophila, mice	None reported	II/III	Safety concerns, delivery challenges, irreversible effects

Abbreviations: HFD, high-fat diet; CKD, chronic kidney disease; T2DM, type 2 diabetes mellitus; GqDNVs, *Lycium barbarum*-derived nanovesicles; CL-EVs, *Allium tuberosum*-derived extracellular vesicles; PK/PD, pharmacokinetic/pharmacodynamic. * Evidence level classification: Level I—human studies (RCTs or observational) for muscle atrophy outcomes; Level II—in vivo animal models; Level III—in vitro/ex vivo mechanistic studies. For interventions with multiple study types, levels are cumulative (e.g., I/II/III). Most interventions remain at preclinical stages (Level II/III).

## Data Availability

No new data were created or analyzed in this study.
